# Book Reviews

**Published:** 1824-02

**Authors:** 


### CRITICAL ANALYSIS
OF
### ENGLISH AND FOREIGN LITERATURE
RELATIVE TO THE VARIOUS BRANCHES OF
### Medical Science.

Quæ laudanda forent, et quæ culpanda, vicissim

Illa, prius, cretâ; mox hæc, carbone, *notamus*.—PERSIUS.

### DIVISION I.

### ENGLISH.

**Art. I.**—*Medico-Chirurgical Transactions, published by the Medical and Chirurgical Society of London.* Vol. XII. Part II.—(Concluded from page 73.)

6. *An Essay on the Proximate Cause of the Disease called Phlegmasia Dolens.* By David D. Davis, m.d.

It is remarkable that the diseases to which women are subject in childbed seem to excite more contrariety of opinion than any other set of complaints, equally common. To what this circumstance may be owing is, perhaps, of less importance to know, than it is to point out the means by which these discrepancies may be removed, and more satisfactory opinions established. There are too many practitioners in this department who labour for their daily bread, and who are too much concerned in the emoluments of the trade, to have much interest in the improvements of the science; and hence investigations likely to facilitate this latter object have been confined to a comparatively limited proportion of individuals. From this there unavoidably resulted a lack of post-mortem examinations, and, consequently, of correct pathological views: hence much of the confusion which has resulted from the general and indiscriminating term "puerperal fever:" and hence, likewise, the variety of speculations, dignified with the name of theories, respecting phlegmasia dolens. For our own parts, we confess that we have perused the paper before us with considerable satisfaction, because, whether Dr. Davis be correct or not in his views, at least he is in the right road,—viz. that of rigorous pathological examination, which, by directing the attention of others to this investigation, may be expected to lead to satisfactory results.

Various are the opinions which have at different times been thrown out with regard to the nature of this affection.—Mauriceau thought it depended on a retention of humors, which should have been evacuated by the lochia; another opinion was, that it arose from a metastasis of the milk, and hence the name of *milk leg*. Mr. WHITE, of Manchester, regarded it as consisting in some obstruction, or other morbid condition, of the lymphatics; and Dr. HULL made it out to be "an inflammatory affection, producing suddenly a considerable effusion of serum and coagulable lymph from the exhalants into the cellular membrane of the limb. The seat of this inflammation he believed to be in the muscles, cellular texture, and lower surface of the cutis; but it does not appear that his opinions were grounded on any examinations after death of parts, concerning which he laid down, what Dr. Davis calls, this "very capacious theory of a proximate cause of disease." We now come to the proximate cause of the author before us, on which subject he shall speak for himself.

"In the development of these facts, it will be my object to prove, or at least to attempt to prove, that the proximate cause of the disease called phlegmasia dolens, is a violent inflammation of one or more of the principal veins within and in the immediate neighbourhood of the pelvis, producing an increased thickness of their coats, the formation of false membranes on their internal surface, a gradual coagulation of their contents, and occasionally a destructive suppuration of their whole texture; in consequence of which, the diameters of the cavities of these important vessels become so greatly diminished, sometimes so totally obstructed, as to be rendered mechanically incompetent to carry forward into their corresponding trunks the venous blood brought to them by their inferior contributory branches."

To substantiate this view, some cases are given, with their dissections: of these we subjoin an abridged account.

I. February 7th, Caroline Dunn, ætat. 21, of weak constitution: a severe labour, which lasted twenty-seven hours; some hemorrhage both before and after delivery; placenta artificially removed. Next and following days, nothing remarkable; some soreness of the vagina. 13th. Slight fever, constipation, labia inflamed, swelled, and œdematous; copious yellow discharge from the vagina, as thick as cream, but without fœtor. 17th. Better; inflammation subsided, discharge diminished, bowels relaxed. 21st and 22d. Better. 26th. Worse; left leg and thigh swollen; pain in the groin, skin hot, no pitting on pressure; bowels costive, much fever. 28th to March 2d. No better; leg pitted on pressure; general depression, no pain. 3d. Total insensibility. 4th. Died at noon. Mr. Lawrence examined the body, and gives the following account of the appearances which presented themselves:

"The left lower extremity presented an uniform œdematous enlargement, without any external discoloration, from the hip to the foot. This was found, on further examination, to proceed from the ordinary anasarcous effusion into the cellular substance. The inguinal glands were a little enlarged, as they usually are in a dropsical limb; but pale coloured, and free from the slightest sign of inflammation. The femoral vein from the ham upwards, the external iliac, and the common iliac veins, as far as the junction of the latter, with the corresponding trunk of the right side, were distended and firmly plugged with what appeared externally a coagulum of blood. The femoral portion of the vein, slightly thickened in its coats, and of a deep red colour, was filled with a firm bloody coagulum closely adhering to the sides of the tube, so that it could not be drawn out. As the red colour of the vein might have been caused by the red clot every where in close contact with it, it cannot be deemed a proof of inflammation. The trunk of the profunda was distended in the same way as that of the femoral vein; but the saphena and its branches were empty and healthy. The substance filling the external iliac and common iliac portions of the vein was like the laminated coagulum of an aneurismal sac, at least with a very slight mixture of red particles. The tube was completely obstructed by this matter, more intimately connected to its surface than in the femoral vein; adhering, indeed, as firmly as the coagulum does to any part of an old aneurismal sac. But in its centre there was a cavity containing about a tea-spoonful of a thick fluid, of the consistence of pus, of a light brownish-red tint, and pultaceous appearance.

"The uterus, which had contracted to the usual degree at such a distance of time from delivery, its appendages and blood-vessels, and the vagina, were in a perfectly natural state. There was not the least appearance of vascular congestion about the organ, nor the slightest distention of any of its vessels. Its whole substance was, on the contrary, pale, and the vessels every where contracted and empty.

"The state of the abdominal cavity and its contents was perfectly natural.

"That the substance occupying the upper part of the venous trunk, and the fluid in its central cavity, had been deposited there during life from inflammation of the vessel, does not admit of doubt. I am also decidedly of opinion, in consequence of its firmness and close adhesion to the vein, that the red coagulum in the femoral vein was the result of a similar affection extending along the tube; and that the passage of blood through it, in the whole track submitted to examination, must have been completely obstructed before death."

This dissection is illustrated by an extremely well-executed coloured plate.

II. Mrs. C. died *instantly*, while in the act of raising herself from the recumbent to a sitting posture, having been put to bed six weeks before of her second child. On the day after her delivery, she had been attacked with peritoneal inflammation, which yielded to free bleeding, both general and local. About ten days after, she complained of deep-seated pain in the groin, and along the great vessels of the thigh; the limb was swelled, and very painful. These symptoms were subdued, within about a week, by means of leeches and blistering, so that she was able to move the limb without any pain. From this period up to the time of her death, the convalescence had been rapid and satisfactory.

*Dissection.*—External appearance, and cavity of the chest, healthy. In the abdomen, particularly at the upper part, adhesions between the parietes and the contained viscera, being the results of old inflammation: the rest of the abdominal, as well as the pelvic viscera, were all healthy. The left iliac vein, including about half an inch of the upper part of the femoral vein, was strongly adherent to the cellular texture forming its natural bed; its parietes were unnaturally thick, and the internal coat studded in several places with coagulable lymph, particularly just under Poupart's ligament; the calibre of the vessel was diminished by about one-half; the inguinal glands were not diseased.

III. This case is related by Mr. OLDKNOW, of Nottingham.—Jane Elliot had an easy labour, and did well till the twentieth day after delivery, when she was seized with diarrhœa, which was epidemic at the time. This was checked by means of astringents, but considerable fever continued; and the purging returned on the thirtieth day, at which time the left lower extremity became swollen and painful. She died on the thirty-fourth day!

*Dissection.*—The femoral vein one-third down the thigh, and all the iliac veins, much enlarged, and containing layers of coagulated blood, similar to that found in aneurism; along with this was a grumous fluid, mixed with air, which almost obliterated the cavities of the veins. These appearances, in a lesser degree, extended along the vena cava, as far as the renal veins. The coats of the uterus highly inflamed, and intimately attached to the surrounding parts. The absorbent glands and vessels slightly enlarged as high as the lumbar region.

IV. Mrs. L., of delicate and irritable constitution, delivered 2d of July. Did well till the seventh day, when she appears to have caught cold: she was seized with violent rigor, followed by pain in the chest, which rapidly increased and became very severe. She was freely bled, leeched, and blistered, by which the pain in the chest was nearly removed, but without any corresponding abatement of fever. Symptoms of phlegmasia dolens came on the same evening, and she died on the 23d.

*Dissection.*—The pleura costalis of the left side slightly inflamed, with an effusion of some coagulable lymph, and about six ounces of serum. Abdominal viscera healthy. The left lower extremity considerably enlarged throughout its whole extent, with evident fullness of the labium of the same side. Iliac veins of either side turgid, but presenting no external appearance of disease, and being entirely free from attachments to the contiguous parts. Inguinal glands natural. "On making a careful incision into the left external iliac, it was found to contain a coagulum of blood of firm consistence; but not, at that part, adherent to the internal surface of the vein. Upon examining, however, the common iliac portion of the vessel, adhesion of the same column of coagulum had obviously taken place. * * * The left internal iliac was greatly inflamed, and its diameter was so much contracted by the morbid thickening of its parietes, that it was rendered almost impervious. The right iliac vein, including both its common and external portions, was distended with a similar coagulum."

Dr. Davis alludes to two cases given in the Journal de Physiologie for January 1823, in both of which coagula were found in the iliac veins.

Dr. Birkbeck has likewise communicated to the author the case of a lady, who was affected with swelling of the lower extremity, apparently from disease of the viscera within the pelvis, she not having been parturient for several years: in this instance Dr. Birkbeck could trace the vein along the thigh into the groin, greatly enlarged, indurated, and having its regular form interrupted at intervals, probably marking the site of the valves.

These cases are followed by many interesting remarks, calculated to give plausibility to the opinions set forth by the author. It is stated, that the seat of the primary pain in this complaint corresponds to the situation of the morbid appearances described; and that all veins subject to much pressure or enlargement during pregnancy, appear predisposed to inflammation on the sudden removal of these states. The fact of phlegmasia dolens seldom attacking the same individual more than once, is explained on the supposition that the great veins within the pelvis, being obliterated, are not capable of again taking on a similar disease. This leads, as a necessary consequence, to the discovery of some other means of carrying on the circulation, and this is supposed to be effected by an extensive system of anastomosis: accordingly, the author informs us that, in many cases, the superficial veins, "sometimes the very finest cutaneous veins," may be seen enlarged, and forming clusters of varices, which are peculiarly large and numerous on the hips and abdomen. The process of establishing this circuitous circulation is tedious, occupying from five weeks to as many months; and, where this latter period is required, the patient seldom regains perfect health.

Admitting the justness of this pathology of phlegmasia dolens, the first indication, of course, is to subdue the inflammation of the veins, so as to prevent, if possible, their obliteration, and all the unpleasant consequences resulting therefrom. The author remarks that, considering the supposed seat of the diseased action and the concomitant fever, general bleeding might naturally be regarded as the speediest means of fulfilling the indication laid down above: so far, however, is this idea from being confirmed by the results of his practice, that "it has completely disappointed expectation" in every case where it has been tried. Phlegmasia dolens, he further remarks, frequently occurs in exhausted states of the constitution, when general blood-letting could not be practised without hazard; while the nature of the complaint itself (supposing his view of it to be correct,) is such as to "rob the general system of a large proportion of its blood, by locking it up in the affected extremity." Hence the faintings, &c. are conjectured to arise. Under these circumstances, the author has recourse to, and strongly recommends, the application of leeches to the parts, followed up by a large blister; these measures being varied and repeated according to circumstances. When it is his object to reduce arterial action, and he feels doubtful of the permanency of the operation of the means above mentioned, recourse is had to digitalis in frequent and large doses. Dr. Davis recommends two grains of the powder of digitalis, as prepared by Mr. Battley, to be given *every two, or at furthest every three, hours.* Thus administered, it may be persevered in till the patient has taken from twenty-five to thirty grains: it should then be given more slowly, and suspended as soon as any of its peculiar effects are produced. We are not aware of the peculiarity of Mr. Battley's preparation here specified, but, with respect to digitalis in general, we must be allowed to view this recommendation with some distrust. Either Dr. Davis rates the power of this drug in checking the inflammatory action too high, or we have been peculiarly unfortunate in our own experience upon this point. But we are not disposed to part with Dr. Davis with any expression of dissent, and beg in conclusion to say, that his theory is better supported than many of our medical doctrines which pass current as correct; although we are inclined to suspect that similar appearances, to a less extent, may frequently be found in women who die soon after their delivery,—without the presence of *phlegmasia dolens.*

7. *On the Effects of Stricture of the Urethra, particularly of the Sacculated State of the Bladder; with an Inquiry into a Mode of Treatment to avert this latter Consequence of Stricture, which is often Fatal.* By JOHN SHAW, Esq.

Stricture of the urethra is one of those distressing complaints which form the daily vexation of many patients, and, we might almost say, affords the maintenance of many surgeons; every information coming from a respectable quarter must, therefore, be regarded as highly deserving of attention. Mr. SHAW appears to have devoted considerable study to the prosecution of this subject, having formerly published some valuable Notes to

Mr. Bell's work on the Urethra; and the object of his present paper is to establish some points connected with the pathology of the urinary organs, which are generally too little attended to. The facts alluded to are given in the author's words.

"1st. I have not, in more than a hundred dissections which I have made of diseases of the urethra, seen a stricture, or narrowing of the canal, posterior to the ligament of the bulb: nor have I been able to find one example of stricture beyond this part, among those preserved in the College Museum.

"2d. In almost every instance where a narrow stricture has existed for some time, in any part of the urethra anterior to the ligament of the bulb, I have found the membranous and prostatic portions dilated to three or four times their natural size.

"3d. The ducts of the prostate, which are naturally very small, are always more or less enlarged when there has been a stricture, or a long-continued irritation of the canal.

"4th. When such a stricture as causes occasional retention of urine has existed for some years, the bladder is found to be not only thickened, but often at the same time sacculated."

The importance of these propositions will be acknowledged by all experienced surgeons: indeed, if they be correct, (and the evidence before us is strongly in their favour,) they give rise to some practical hints of considerable value. If the first holds good in the great majority of cases,—and, so far as the author's observations have extended, it has been universal,—it is obvious that if, on introducing an instrument into the bladder, it is obstructed at any point posterior to the ligament of the bulb, such obstruction must arise from some other cause than stricture, and consequently demands a different method of cure. The same remark applies with even more aptitude to the second observation, and leads to the rule laid down by the author, that, on meeting with an impediment beyond the ligament of the bulb, we ought not to attempt to overcome it by force. The enlargement of the ducts of the prostate, forming the third proposition, is one generally known, though not, perhaps, sufficiently attended to. The danger of the point of the instrument entering the mouth of an enlarged duct, is obvious; and the consequence of pushing the bougie further must unavoidably be the formation of a false passage. Several of the preparations in Great Windmill-street show the instrument may be even forced into the posterior part of the dilated bladder.

Mr. Shaw next proceeds to examine anatomically that portion of the urethra which is surrounded by the ligament of the bulb. The principal circumstances insisted upon are, the abruptness with which the canal has become narrow, and the direction being suddenly changed: thus giving rise, even in the natural state, to a degree of mechanical obstruction not experienced elsewhere. To these natural impediments are to be added the effects produced by the contraction of the muscles surrounding this part of the urethra. All these difficulties are increased when the membrane lining the passage is inflamed, even though but in a slight degree, as a spasmodic action is brought on as soon as the instrument comes upon the diseased part: these "may produce so complete an obstruction to the entry of an instrument, as to give rise to the idea of the presence of stricture." Another important fact pointed out by the author, and which suggests a useful caution, is that the bougie may be "so cut or indented," as to assume the appearance of having been thus marked by a stricture: this arises from its being pressed against the lower edge of the ligament.

The sacculated state of the bladder is so common, that Mr. Shaw informs us he has come to the conclusion, that, "if a very narrow stricture has existed for a certain time, and the patient has suffered occasional attacks of retention of urine, a sac has probably formed." The precise symptoms by which the existence of a sac may be detected, it is not easy to point out; but the author seems to regard irritation at the back part of the bladder, or between it and the rectum, especially when it occurs after voiding urine, as one of the most characteristic. The formation of a sac bladder, and still more a sacculated condition of the prostate, give rise to a fistulous communication between the rectum and bladder, more frequently than might be supposed from the comparative silence of practical writers on this point. The existence of a stricture does not appear to be necessary for the formation of this false passage, as the prostate is "very liable" to become sacculated without any such obstruction. In inquiring into the means of alleviating or averting these distressing and untractable accidents, Mr. Shaw supposes the following case:

"A patient has had a stricture near the bulb for several years; every plan of treatment, as by bougies, caustic, and forcing, has been tried, but with so little success, that now the smallest bougie cannot pass the stricture. The patient has frequent attacks of inflammation of the bladder, and the water dribbles from him, or is passed only drop by drop.

"When a patient is in such a condition, what have we hope for, and what are we to dread, if something decided be not done to free the stricture, and, at the same time, to relieve the bladder from the constant irritation under which it suffers?"

It is reasonably presumed, that the ordinary routine of practice for the cure of such cases has been employed without avail, and all we can look for now is to allay the irritation; or, failing in this, that nature may take the case into her own hands, and form a fistulous opening in the perineum, as the least evil, and the best method of preserving life. But, bad as this condition is, let us inquire the state of the patient before its occurrence: he has a stricture, which prevents any instrument from passing, and a diseased condition of the bladder. Now, supposing him to catch cold, or to run into any dissipation, no matter how slight, and the probability is that complete retention of urine comes on, requiring that the bladder be evacuated by operation. But, supposing that this striking mischief does not occur, and that, by prudence or good fortune, the patient escapes from an attack of retention of urine, still the constant irritation of the bladder generally gives rise to a sacculated condition of that organ, to disease of the prostate, and a train of evils by which the constitution is slowly, but inevitably, exhausted. "Under such circumstances, (asks Mr. Shaw,) are we not entitled to say, that something decided should be done, with a view to remove the stricture and relieve the bladder?" Granting that *something* should be done, the next and most important question is, what this something ought to be? Forcing the stricture is decided against, because the diseased part is generally much firmer than any other portion of the urethra, and therefore, of all others, it is the least likely to yield. Puncturing the bladder is much more rational practice; but this, unfortunately, only gives temporary relief. We shall now let Mr. Shaw speak for himself.

"Seeing the many dangers to which a patient, in such a condition, is liable; and having, by experience, found that, when the urgent symptoms (which we must expect) do come on, prompt measures must be used, or our patient will be lost; are we not entitled to inculcate the propriety of performing an operation while the parts are yet comparatively in a favourable state? The proposal is the more encouraging, as the operation, if dexterously performed, is not severe, nor attended with any danger; and it is moreover one which will probably not only afford immediate relief to the bladder, but also, if not the means of restoring the patient to perfect health, put him into a condition of much greater ease and comfort than could be expected, if his disease were to terminate in what we should consider its most favourable natural issue.

"The operation is not severe,—indeed, much less than what almost any patient will cheerfully submit to, in the mere hope of being relieved from the inconvenience of a fistula in perineo. It is merely to cut through the stricture, to introduce a catheter from the glans, and endeavour to make the urethra entire, by allowing the wound to granulate over the catheter."

The only difficulties which suggest themselves to the author in performing this operation, are—1st, that we may introduce the catheter into one of the false passages previously made, instead of into the strictured portion of the urethra: great care must, of course, be taken to avoid this, as the instrument would not guide the incision to the proper point; and, 2dly, that, after the stricture has been cut through, it may not be easy to direct the catheter into the proper canal, as there may be various false openings: to obviate this, the patient should either have retained his urine before, so as to be able to void it now, or else the further progress of the operation must be postponed until the bladder be filled again. After the wound has closed over the catheter, the passage must be kept free for a considerable time by bougies. It seems fairly enough urged by the author, that, if the wound does not readily close, still the patient would be in no worse a condition than he would be placed by the most fortunate accident which could occur, if left to the surgery of nature. This reasoning must be admitted as just, provided it be correct that the operation proposed is unattended with danger. We do not mean to say that, if it should be accompanied with hazard, we are therefore to condemn it; but we should then have to weigh the dangers, and choose the least; this, we believe, will for the most part be favourable to Mr. Shaw's views.

The author alludes to one successful case, and another (perhaps the same?) is detailed by Mr. ARNOTT in the volume before us. We have given the operation, as described by this gentleman, at length, and are therefore less inclined to dwell upon it now.

### 8. *Illustrations of the Medical Properties of Quinina*, By JOHN ELLIOTSON, M.D. &c. &c.

Dr. ELLIOTSON, who has published some interesting remarks on the effects of various medicines, as prussic acid, pulvis antimonialis, &c. has recently tried some experiments with the sulphate of quinina, the account of which is preceded by a brief notice of the observations made on it in France. It appears that Dr. CHOMEL gave this substance in thirteen cases of intermittent fever; of these, ten were cured, and, in three unsuccessful cases, the cinchona itself was given without avail.* M. DOUBLE tried it in six cases, in all of which it proved successful. "Instances of the equally successful exhibition of the sulphate of quinina, by M. VILLERMÉ,† MAGENDIE,‡ FALLOT,§ and DUPRÉ,|| are recorded.

The first case related by Dr. Elliotson is one of typhus, in which the medicine is supposed to have been eminently successful.

---

* *Journal de Pharmacie*, 1821.

† *Bulletin de la Société Medicale d'Emulation*, 1821.

‡ *Journal de Physiologie.*

§ *Journal Complémentaire*, 1822.

|| *Journal de Physiologie*, 1822.

"A poor Irish woman, half starved and flooding, was brought into St. Thomas's, labouring under severe typhus, on the 19th of June. She was supported by plenty of beef-tea and milk; the epigastrium, forehead, and occiput, were blistered; and hyd. cum creta was prescribed in doses of one scruple, and sometimes two scruples, every six hours, till the mouth grew sore. The delirium and stupor were entirely consumed, and the tongue became clean and moist, but the debility increased hourly. The face became ghastly, and the body sunk lower in the bed. I ordered three, and soon five, grains of the sulphate of quinina, to be given every six hours, and the diet to remain as before. A striking amendment was observed the next day, and she speedily recovered. After being convalescent some time, the medicine was omitted; but, when I thought of discharging her, she suddenly relapsed into extreme prostration of strength, passed her urine and fæces again involuntarily, and grew delirious; but the tongue remained clean and moist. The two blisters to the head were repeated, and the sulphate ordered as before; milk and beef-tea, *ad libitum*, continuing to be her diet. The amendment was not so sudden, but, from the first day of recurring to the medicine, the debility ceased to increase; in a few days she clearly gained strength, and was soon convalescent. After taking the full diet of the house, and a pint of porter daily for two or three weeks, she was discharged perfectly strong and well."

The three next cases are intermittent fevers: five grains of the sulphate of quinina were given every six hours, in the form of pill. In the two former, the patients got well very rapidly; in the latter, it required rather more than a fortnight to eradicate the disease.

Dr. Elliotson next tried experiments with the simple quinina, and the result of these was highly satisfactory; eleven cases of intermittents being detailed, in all of which it proved successful. Dr. Elliotson has never observed the slightest unpleasant effects, either from the quinina or its sulphate, although he has frequently given them every day for several weeks, and has pushed the dose to the extent of a scruple in twenty-four hours. Ten grains for a dose was found to occasion vomiting; and five grains of the sulphate, every six hours, is regarded by Dr. Elliotson as the largest dose that can be necessary.

9. *An Account of two Cases of Biliary Calculi, of extraordinary Dimensions.* By T. BRAYNE, Esq. of Banbury. Communicated by Mr. TRAVERS.

The first of these occurred in an old lady, aged fifty-five, of a spare habit and melancholic temperament, and nothing remarkable is presented by the case, except the description of the calculus itself. This resembled a pigeon's egg in shape, but was rather larger and more flattened; the colour was a light yellow, variegated with brown, and it much resembled a compound formed of soiled fragments of spermaceti; its weight was

162 grains (next day); the transverse circumference, at the widest part, $3\frac{2}{8}$ inches; the long diameter, $1\frac{3}{8}$ of an inch; short diameter, $1^{8}$ of an inch. This calculus is different from the common appearances of such bodies, in wanting the lanunated texture, and in containing a larger proportion of adipocire. A year and a half after she had passed this calculus, the patient died of hydrothorax, and it was found, by post-mortem examination, that "the liver was of unusual size and appearance, in respect to colour, and seemed to vary from its natural structure in nothing but in being rather more close and solid in its texture, and more resisting to the pressure of the finger. The cystic and hepatic ducts were of the usual dimensions, but the gall-bladder itself was smaller, and very much thickened, containing only a little pale unhealthy bile. It had contracted a long adhesion, about the size of a shilling, to the duodenum, close to the pylorus. There was no uncommon appearance of vascularity. On removing these parts from the body, a communicating aperture, large enough to admit a crow-quill, was discovered in the centre of the adhesion. The duodenum and gall-bladder were afterwards inflated from the hepatic duct, and preserved." From this account it is obvious that the gall-stone did not effect its passage by dilating the ducts, but by ulcerating its way into the duodenum.

The second patient was likewise a female, and aged sixty-five. After severe symptoms, consisting in constipation, vomiting, pain of the abdomen, restlessness, &c. she passed two biliary calculi,—one of a "flat square shape, with its angles rounded, and the two sides of the greatest surface considerably hollowed out, as if compressed, when soft, by some convex body;" it weighed 176 grains. The other resembled half a large pigeon's egg, and the weight was 159 grains.

10. *On the Destruction of the Fœtal Brain.* By Mr. HAMMOND.

A young woman had the pelvis too narrow to admit of the fœtus passing by the natural means: it became necessary to diminish the size of the head. An opening was made near the fontanelle,—a portion of bone removed,—the fingers introduced,—the cerebrum completely broken down, and two ounces of it taken away; the fœtus was then quickly extracted. It cried heartily, breathed well, and apparently with the natural degree of strength: for twelve hours the functions were performed in the usual healthy manner; after this it gradually became weaker, and died at the end of ten hours. The bleeding from the wound, though not violent, had never ceased, and the child was supposed to sink from the hemorrhage. On examination after death, the dura mater was found considerably torn; the cerebrum broken down into a mass, devoid of organization; neither the cerebellum, medulla oblongata, nor spinal marrow, were injured. There is some indistinctness with regard to the dates in this case. We are told that the child cried, &c.; and that, for twelve hours, the functions were carried on in the usual manner; next, that the child began to sink, and continued ten hours in this state, and was slightly convulsed during the last two: from which we inferred that it had lived twenty-two, or at the utmost twenty-four, hours; but it appears that life was prolonged to forty-six.

11. *Case of Bronchocele.* By H. S. Roots, M.D. Physician to the Carey-street Public Dispensary, &c.

A young lady, of scrofulous diathesis, had been affected with enlargement of the thyroid gland for two years, which had been rapidly increasing during three or four months, being at times attended with some uneasiness: both lobes were enlarged, and the tumor resembled an orange flattened. Leeches were applied externally twice a-week, and carbonate of soda given internally, for three months, without avail; after which, leeches were applied once a-fortnight, and three drachms of burnt sponge ordered three times a-day. This plan likewise was employed for three months without benefit. The linimentum saponis was rubbed twice a-day, in small quantity, and with gentle friction, into the swelling, for a like period; the burnt sponge being continued. This likewise failed.

The next means adopted was rubbing into the tumor, night and morning, the size of a garden-bean of the following ointment:—

℞. Potassæ Hydriodat. gr. xxxiv.

Ceræ albæ, ʒij.

Adipis Suillæ, ℥jss. M.

This was persevered in for five weeks, at the end of which time the circumference of the tumor was found to have diminished three-quarters of an inch. The proportion of iodine was now increased to forty-four grains, and the use of the ointment continued; but, after it had been applied four or five times, inflammation came on, and it was consequently omitted for a week. No inflammation being induced by its re-application, the iodine was gradually increased to fifty grains, in which proportion she continued to use it till the latter part of July, (she originally began it in January,) at which time the tumor on the left side had disappeared, while that on the right side was materially diminished. The tincture of iodine was ordered in doses of gtt. xx. ter die; but, this disagreeing with the stomach, it was taken only twice a-day, and the ointment continued, the quantity of iodine being augmented to fifty-six grains. By November of the same year, the swelling was entirely removed.

12. *On the Dilatation of the Male Urethra by Inflation, for the Extraction of Calculi from the Bladder, as practised in Egypt nearly* 250 *years ago.* By ROBERT MASTERS KERRISON, M.D.

This paper consists principally of a long extract from the work of PROSPER ALPINUS, "De Medicinâ Egyptiorum," describing a method of extracting small calculi, upon a principle similar to that which has lately been adopted in this country. FABRICIUS HILDANUS has likewise recommended the inflation of the male urethra for similar purposes.

13. *Case of Stricture of the Urethra treated by Incision.* By JAMES ARNOTT, Esq.

A married man, aged forty-nine years, of spare frame and temperate habits, had suffered under stricture of the urethra for fifteen years, during the last four of which it had been impossible to introduce the bougie. When he consulted Mr. ARNOTT, he was called upon every hour to void his urine, which came away *guttatim*. The bougie was stopt towards the bulb of the urethra, nor could the smallest instrument of this kind be introduced. Caustic was applied without avail; and it was resolved (after consultation with Mr. SHAW,) to cut down upon the stricture.

"Owing to a contraction of the urethra an inch and a half from its extremity, a catheter under the middle size only, could be passed down to the obstruction at the bulb; and, as its point could be but obscurely made out in the perineum, the external incision was made rather free. On reaching it, and making an incision in the urethra anterior to the obstruction, the point of a very small grooved probe was guided into the aperture, and pushed on towards the stricture, into which it entered with little difficulty, and was afterwards felt to have entered the bladder. Upon this a bistouri was run down, and the strictured portion divided, occupying, if one might judge from the sensation, not so much as a quarter of an inch in length. The probe being kept in its place in the urethra, the catheter was immediately carried onwards with great facility into the bladder, and upwards of a pint of urine drawn off; although, about ten minutes before the operation, the patient, not being able to resist the pressing call to make water, passed a small quantity, and had, as he supposed, completely emptied his bladder.

"No unfavourable symptom occurred. On the fourth day the catheter was withdrawn; for, when the patient was making water through it, the urine passed partly by the sides of the instrument, between it and the urethra. It was replaced by a large-sized one of elastic gum; but in two days afterwards this was taken out, as it seemed to create irritation, and a silver one, of a size fuller, passed in its place; which again, in two days more, gave way to one of the largest size. The wound healed favourably: for the first five or six days its lips were merely moistened with urine; by the end of a fortnight it was quite closed. The catheter was then withdrawn during the day, the patient now making water in a full stream: the instrument was, however, continued to be worn at night for another week, when it was left off, and a bougie afterwards passed only occasionally. In the course of the first eight days of wearing the catheter in the urethra, the induration surrounding the contraction in the canal, an inch and a half from its extremity, had entirely dispersed, and the largest-sized catheter passed with facility."

14. *An Account of a rare Case of complicated Labour, from Locking of the Heads of Twins, &c.* By JOHN ALLAN, Esq.

A small spare woman, thirty years of age, was taken in her third labour, and at the end of eight hours the membranes were found entire, but apparently empty of the liquor amnii; the os uteri was obliterated; the left knee presented, and with it the vertex of the head. An attempt was made, but without success, to push up the limb, so as to allow the head to descend; the membranes were then ruptured with some difficulty, (owing to their unusual toughness,) and the fore-finger introduced so as to bring down the knee; upon which the head was felt to retire upwards. The right arm was wedged between the occiput and the symphysis pubis, and was not disengaged without difficulty.

"No pulsation being perceptible in the cord, I was proceeding, in the usual manner, to introduce two fingers of my left hand into the child's mouth, for the purpose of bringing the chin down to the breast, and hastening the delivery, when I discovered that the hollow of the sacrum was occupied by the head of another child, of which the body was still above the brim of the pelvis. The face of this second child was towards the sacrum, and its occiput was closely applied to the throat of the first-mentioned child. The back of the neck of the latter was closely applied to the symphysis pubis of the mother, and its face to the back of the neck of the child whose body remained within the uterus. With the heads thus situated, it would have been impossible, even had the firm contraction of the uterus been out of the question, to have pushed upwards the one next to the sacrum, without carrying the other before it; and every attempt to extract that which was next to the pubes had the effect of pressing the other so forcibly downwards, as to threaten a rupture of the perineum. In this dilemma, I was at first rather doubtful what course to pursue, the case, so far as then known to me, being without a parallel; but, after a little consideration, I resolved upon a mode of completing the delivery, which will be described presently, and which may, perhaps, be advantageously adopted on similar occasions in future. Owing to the smallness of the children, it proved unnecessary in the present instance, both heads having been simultaneously expelled from the pelvis by one powerful parturient effort, without any assistance from art. Both children were irrecoverably dead. They appeared to be six weeks or more before the full period. The mother had a smart attack of hysteritis, marked by uterine pain and suppression of the lochia, and accompanied with great excitement of the vascular system and severe intolerance of light. Three bleedings, amounting together to seventy-six ounces, and free purging, with other appropriate means, removed these symptoms, and she recovered perfectly."

In the Edinburgh Medical and Surgical Journal for January, 1822, a similar case is detailed; and another in the Journal de Medecine for 1771. Mr. Allan suggests that, in such unfortunate circumstances, the body of the child which has passed the os externum might be detached, and the head pushed further above the brim of the pelvis, while the other head might be extracted by the forceps, provided the natural efforts were insufficient. After this, the head which had been detached might be removed by instruments.

**15.** ***A Case of Ascites, connected with Utero-Gestation, successfully treated by Operation.*** **By George Langstaff, Esq.**

This is a very interesting case. A lady, aged thirty-nine, who had given birth to eight children, again became pregnant. She soon became unusually large, and very uncomfortable: at the fifth month she had become so large as to lead to the suspicion that the pregnancy must be further advanced than she had supposed. By the end of the sixth month, the pain and distention had become so distressing as to require general and local bleeding, and a blister to the abdomen. It now became evident, from the œdema of the lower extremities, as well as from fluctuation in the abdomen, that dropsical effusion had taken place. Calomel, digitalis, and squill, were tried without relief. Dr. Farre was now consulted, in order to determine whether labour should be brought on, or the patient be tapped: the former plan was agreed upon, and this decision afterwards confirmed by Dr. Davis. The unwieldy state of the abdomen becoming every day aggravated, the liquor amnii was at length let off: it was in small quantity. Next day the symptoms were more distressing than before, and, as labour did not come on, it was resolved to draw off the water by tapping.

"On the 20th of March, I cut down to the peritoneum, about two inches below the umbilicus, then carefully perforated that membrane with a moderate-sized instrument, such as is commonly employed in performing the operation of paracentesis abdominis, taking especial care to introduce the point of the trocar only a short distance beyond its shoulder, that the uterus might not be injured; the trocar was then withdrawn, and the canula pushed a little further into the abdomen. The fluid, which flowed very freely, was transparent; when about ten pints of it had flowed, the stream was checked by the anterior part of the uterus coming in contact with the point of the canula: this occasioned so much pain as to oblige me to withdraw it, hoping that, by means of a bandage round the abdomen, assisted by slight pressure on each side with the hands, the remainder of the fluid might be evacuated. In this, however, I was disappointed, as the patient could not bear the necessary pressure. I therefore introduced a moderately large, smooth, soft, elastic-gum catheter through the opening, which passed downwards for several inches between the anterior part of the peritoneum and uterus, and thus drew off the remainder of the fluid. The whole amounted to twenty-five pints. About eight hours after the operation, pain was complained of over the whole of the abdomen; the patient was restless, skin hot; pulse 120, not full but wiry. Twenty-four ounces of blood were drawn from a vein in the arm, which was considerably more inflamed than the blood usually is when taken during pregnancy. Saline aperient medicines were prescribed, and five grains of calomel, with the same quantity of the extract of hyoscyamus at bed-time."

She had twenty leeches applied on the 21st, and thirty ounces of blood were taken from the arm on the 22d. On the 23d, she was considerably better; and in the evening of the same day she was delivered of a dead child, four hours after the commencement of the labour. The fœtus had been dead several days, and did not appear to have advanced beyond the seventh month. The patient did well.

16. *Further Account of the Extraction of Calculi from the Bladder, without the Use of any cutting Instrument.* By Sir A. COOPER, Bart. F.R.S.

This paper contains some additional instances of the successful application of the instrument for dilating the urethra. The first occurred in the practice of Mr. BRODIE, by whom about sixty calculi were removed from the bladder of a gentleman seventy years of age: these were of various sizes, the largest measuring half an inch in one diameter, and five-eighths of an inch in the other. The second case occurred in a patient of Sir GILBERT BLANE's and Sir A. COOPER's: a stone weighing fifty-four grains was extracted with some difficulty, the greatest obstruction being experienced at that part of the urethra which is behind the glans. Sir A. Cooper is of opinion that it would be better to make an incision into the urethra anterior to the scrotum, rather than to employ force in extracting the stone. The third patient was a gentleman aged sixty-six, a mariner, from whom Sir Astley extracted four calculi on the 30th of October, three on the 1st of November, four on the 5th, twelve on the 7th, two on the 11th, and three on the 13th, when no more could be felt, and the symptoms were relieved. Another case is alluded to, in which the author likewise extracted a moderate-sized calculus, and enabled two others to pass.

17. ***On Injuries of the Pelvis.*** By JOSEPH SWAN, Esq. of Lincoln.

Four cases of this nature are related. In the first, the bones of the pelvis were separated at the pubis, and the body of the right bone fractured; its ramus was also broken where it joins the ischium; a considerable portion was completely detached, and denuded of the periosteum. A quantity of blood and urine were extravasated among the muscles in the surrounding parts, and two inches of the urethra completely torn away. The patient died on the seventh day.

In the second, (which took place in a female from being thrown from a gig,) the knee and foot were turned outwards as she lay in bed: on pressing the ilium and moving the limb, a crepitus was perceptible; when out of bed, the foot could be placed flat on the ground, and she could sit without much inconvenience when the heels were put close together. The fracture began about two inches from the anterior superior spinous process of the ilium, and was supposed to extend into the acetabulum. An inequality of crista ilii remained ever after, by which the site of the fracture could be detected. This patient recovered: a tight bandage was at first applied round the pelvis, and a broad leather girdle worn afterwards.

The third case is that of a lady, who "complained of pain in her hips, and felt as if they would break asunder whenever she attempted to walk. She walked with the greatest difficulty, and frequently, in doing this, her limbs were so weak as to cause her to fall down." She recovered under the use of absolute rest and tonics.

Case IV.—A loaded waggon passed over a man, aged twenty-four, during a state of intoxication: some blood was observed to come from the penis, and, on pressing the pubis with one hand, and the ischium with the other, a distinct crepitus could be felt. The urethra was ruptured, and, the parts swelling from the extravasation of urine, an incision was made into the perineum, by which it was evacuated. Every thing that the case admitted of was had recourse to for his relief, but he died on the fourth day after the accident. The following account is given of the examination:

"On opening the abdomen nine hours after death, the peritoneum was sound, but blood had been effused behind it as high as the superior parts of the kidneys. A very small quantity of serum was in the abdomen, otherwise every part covered by the peritoneum was perfectly healthy. The arch of the pubis was quite broken off, and was only kept in its place by Poupart's ligaments. Several other portions, both of the bones of the pubes and ischium, were broken off. The acetabulum of the right side was opened, and matter was contained in it. The mischief was the greatest on the right side. The right sacro-iliac symphysis was fractured. Blood and urine had escaped to the lower part of the thigh, especially about the sciatic nerve. A very large rent was found in the anterior part of the bladder; the urethra was torn completely through. The wound in the perineum looked very well, and no sloughing had taken place in any of the injured parts."

Mr. Swan tried the following experiment:—He divided the symphysis pubis in a rabbit, and then cut out a portion of the os pubis, a twelfth of an inch in width: the edges of the wound were brought together, and healed. The animal recovered, but the hind legs remained much separated. On being killed, the divided portions of the bone were found to be separated full three-quarters of an inch, the space being filled by a strong membrane.

18. *An Account of a Case of Axillary Aneurism, in which the Operation of tying the Subclavian Artery was successfully performed.* By HARRY LEAKE GIBBS, M.D.

This is the case to which we alluded in a former Number, in speaking of Mr. BRODIE's similar operation at St. George's. A cooper, aged thirty-five, had an axillary aneurism, about the size of a goose's egg, for which the following operation was performed:

"January 5th, 1823.—The patient being placed on a table, with his head towards the light, inclining backwards towards the right shoulder, the integuments above the clavicle were pinched, or drawn up, and being pierced with a straight bistoury, presented an incision of three inches in length, parallel to, and a quarter of an inch above, the clavicle. The platysma myoides was thus divided, and the external jugular avoided. This vein causing embarrassment, from its alternate swelling and collapse, I enlarged the incision an inch inwards, and, passing a director under the clavicular portion of the sterno-cleido mastoideus muscle, close to the clavicle, separated it from its attachments. Its contraction afforded tolerable space, and, by means of the finger, the subclavian artery, as it passes over the first rib, was felt, but very faintly, from the state of syncope into which the patient had nearly fallen, at the same time that he had a severe rigor. I delayed the operation ten minutes, covering the poor man with blankets, and giving him cordials. At the expiration of this time, the pulse of the artery had recovered itself, and, being separated by the nail and finger from the connecting parts, a round ligature was passed under it, by means of a stout silver needle, much curved, and fitted to a strong handle: the instrument being passed under the vessel first, the ligature was passed through the eye, and drawn under. I instantly raised the vessel, and, finding that the pulsation of the tumor was thereby stopped, tied it in a double knot, as it passes over the first rib. The wound was then brought together with adhesive plaster, the patient dressed, and put to bed."

The ligature came away on the twelfth day, and the patient did well.

19. *Rupture of the Uterus, and subsequent Recovery of the Patient.* By JAMES POWELL, Esq.

A patient of low stature, and considerably deformed, was taken with slight uterine pains, which continued lingering, and with little effect, from Monday till six o'clock on Thursday evening, when the orifice of the uterus was the size of a crown-piece, with the head presenting. Strong bearing-down pains came on soon after, and lasted with increasing force till eight o'clock, when they suddenly ceased, and were succeeded by "a most excruciating pain of a different kind." This change was attended by anxiety of countenance and other symptoms of serious mischief, and accordingly, on examining *per vaginam*, the child was found to have escaped into the cavity of the abdomen. Dr. DAVIS was sent for, who found that the rupture extended along the whole course of the neck of the uterus on the right side, including the orifice. The delivery was effected by turning, and craniotomy became necessary before the head could be extracted. The patient's strength sunk so much, as to require repeated spoonsful of undiluted brandy during the operation; no hemorrhage nor descent of intestine followed. Sixty minims of Battley's liquor opii sedativus was given, and she ultimately recovered.

20. *Case of a Preternatural Growth on the lining Membrane covering the Trunks of the Vessels proceeding from the Arch of the Aorta.* By JOHN YELLOLY, M.D. &c.

The history of this case is briefly this:—A man, aged fifty-six, suddenly dropped down dead; the heart was found rather larger than usual, both sides being remarkably turgid, particularly the left; the coronary vessels and valves were all healthy; the ascending aorta was somewhat increased in size, and on its inner surface were found irregular scales of ossification.

"The trunk of the arteria innominata, and the trunks of the left carotid and of the left subclavian arteries, were all of them in a considerable degree plugged up with a growth from the lining membrane of the artery, having the same general nature and appearance as the lining itself, and without any ossific deposition. In the arteria innominata, this preternatural growth extended irregularly about an inch up the vessel, the calibre of which was reduced by it to less than one-third of its usual dimension. In the left carotid, it was confined nearly to the opening of the trunk into the aorta; but the orifice was diminished to such an extent, as to admit not more than the passage of a common-sized probe. In the left subclavian, it extended about half an inch up the vessel, the cavity of which it had diminished to the extent of a small slit. No other morbid appearances were observed."

This patient's general health had always been good, with the slight exception that, within the last two years, he had experienced two or three attacks of faintness; from which, however, he speedily recovered.

21. *Abstract of the History of a Case of Strangulated Exomphalos, successfully operated on, fifty Hours after Parturition.* By Mr. GORE.

A lady was put to bed on the 20th of December, and next day was found to labour under strangulated umbilical hernia. The operation was performed by Mr. TRAVERS: sloughing of the omentum took place, and feculent matter was passed in large quantity by the wound. Notwithstanding these symptoms, the wound closed, and the patient recovered.*

---

ART. II.—*Practical Observations on Fever, Dysentery, and Liver Complaints, as they occur amongst the European Troops in India: illustrated by numerous Tables and Cases. To which is annexed, an Essay on Syphilis.* By GEORGE BALLINGALL, M.D. F.R.S. E. Fellow of the Royal College of Surgeons of Edinburgh; Surgeon Extraordinary to the King for Scotland; Regius Professor of Military Surgery in the University of Edinburgh; and one of the Surgeons to the Royal Infirmary of that City. Second Edition.—Edinburgh: Black, 1823.

THE wisdom of Providence has ordained that good shall proceed out of evil, and the last twenty-five years of a most sanguinary contest, carried on in nearly all the different climates of the earth, has fully proved the truth of this remark, as applicable to our profession; for within that period a race of medical men has sprung up, who have investigated, with patience, courage, and success, many of those diseases which, like vultures, follow in the rear of armies, and which cause more real distress, and a larger number of deaths, than the sword of the enemy. This remark applies with equal force to the army and navy medical officers of France, as well as England; and there are few villages, in our own country especially, that are not now supplied from these sources with practitioners of a class infinitely superior to those of the previous generation: so much so, indeed, as strikingly to have diminished the number of great operations formerly transferred from the provinces to the capital, as well as the resort of invalids to town for the purpose of consultation.

In the foremost rank of this meritorious class of men is the author of the volume before us, who informs us, in his preface, that the contents of his book are the result of his personal observation, and that it was written in a state of seclusion from

* The only paper omitted in this Analysis is that of Mr. JACOB on the Eye; a full account of which was given in our "HISTORICAL RETROSPECT" last month.

all intercourse with the medical world, and with a library extremely circumscribed. Now, these are precisely the circumstances which, in our opinion, give works of this description their value: they are the vivid impressions made at the bedside of the sufferer,—made upon one previously well acquainted with the rudiments of his profession, and *fortunately* removed from the possibility of torturing what he sees, to meet the fashionable theory of the day. It is true that a man writing thus may, and does, say what, perhaps, has been said some hundreds of years, or some hundred of times, previously; but there is still the freshness and vigour of truth in all his descriptions, and, as far as the mass of practitioners is concerned, his labours will have all the merit of novelty: whereas, the mere book-instructed physician will know by heart what some venerable ancient has said of this or that particular disease, but, when turned into an hospital, he is totally unable to recognize the original. We do not wish by any means to depreciate the labours of our forefathers, nor would we encourage the professional student to neglect the careful study of the great masters of the art; but we only mean to assert that, when a young man has carefully gone through his probationary studies, he who goes from his closet to the patient's bedside, and from thence to his closet again, will be more likely to fulfil the intent of his education,—that is, the relief of suffering humanity,—than he who can quote Hippocrates, Aræteus, Ætius, or Albucacis, who knows all the symptoms that should be assembled to constitute a particular disease, but who is thrown out if he does not meet with them in the exact order and proportion set down in his book.

The work, of which we are now about to give an account, is nearly a reprint of the first edition of 1818,—that is to say, the alterations are more as to the arrangement than in substance, with some little improvement in the form of expression. Our readers will perceive, from the nature and number of the subjects treated of, and the size of the volume, it was not the author's intention to give an elaborate treatise on these complaints, but merely to dwell upon and illustrate certain points of doctrine, or practice, which had either been overlooked or left unexplained by those who have traversed the same ground.

The first nineteen pages are devoted to some introductory remarks on the unfitness of boys, or very young men, for military service in the East Indies. These observations, though characterised by good sense, and which military as well as medical men now well understand and appreciate, need not detain us, since the principle appears to be acknowledged and acted upon by the ruling powers. We shall only state, that the facts which our author has adduced relative to the second battalion of the Royal Scots, and the register of deaths which is to be found in Appendix No. I., fully confirm his views upon this subject.

The first disease treated of is *fever*, and we quote the first paragraphs.

"Fever does not, in general, occupy the most prominent part in the sick-returns of the Indian army; but, when we reflect upon the number of invalids to be met with in that country, who date the origin of their protracted sufferings from an attack of fever; when we see men travelling from one station to another, and ultimately obliged to return to Europe in search of that health which, previous to the attack of fever, was never known to be impaired; in fine, when we consider the number of visceral obstructions, the consequences of this disease, we are induced to look upon the sequelæ of fever as by far the most formidable part of it, and to inquire whether something has not been deficient in the treatment of its earlier stages. From inquiries of this nature, I have often been led to believe that the treatment which has proved sufficient to prolong a patient's existence, has by no means been competent to the restoration of his health; that the practice which has, in the first instance, barely sufficed to rescue him from the grave, has ultimately been the means of prolonging his distress, and has snatched him from the jaws of death only to give him life upon terms under which it was scarcely worth possessing.

"The object of the following paper, therefore, is to recommend the adoption of a practice in the early stages of fever, which the urgency of the symptoms would not perhaps seem to demand,—to press upon the attention of practitioners the necessity of a vigorous use of evacuations at the commencement of this disease,—and to urge the liberal use of purgatives, blood-letting, and the cold affusion. The first two are particularly adapted, in my opinion, to prevent visceral congestion and ultimate obstructions, so much to be dreaded as the sequelæ of fever; and the last is calculated, when early employed, to put a speedy termination to its progress, and to prevent its fixing upon, and ever afterwards impairing, the functions of important organs."

Our author has not had much experience of those fevers commonly called Jungle, Hill, and Seringapatam fevers; but what he has seen and heard induce him to believe that the sequelæ of the disease are as much to be dreaded as the fever itself, and these sequelæ he believes to be too often the consequence of inert and inefficient practice in the first instance. His own practice in this disease has induced him to form this conclusion; for, during a period of seven years, he had lost only forty-two men by fever, and, of eighty-seven cases of which he had preserved notes, eighty-six terminated favourably. Our author is willing to admit that the character of the fever usually met with in the stations to which his practice was chiefly confined, are not to be compared, in point of violence, with those above alluded to; yet he thinks the difference is more in degree than in kind. The fever most commonly met with by our author, whilst living on the Madras establishment, is what authors term the bilious remittent; the most prominent symptoms of which have been often described, and therefore we do not need here to repeat them. A sense of heat on the skin, absolutely painful to the touch, is occasionally met with; and the irritability of the stomach is usually very great. Dissection shows that the brain, liver, and spleen, are the organs most particularly affected by this disease; and, in all, marks of congestion were most evident, particularly in the liver, the tendency to which (if the patient survive the fever,) too often lays the foundation for abscess or chronic flux. The inference from these remarks is to institute a practice with a view to these after consequences, which the extent of the symptoms would not otherwise seem to warrant. The means he therefore recommends are, indeed, those very generally employed; but the extent to which they are commonly used is not, in his opinion, sufficient.

Dr. B. proceeds to illustrate his meaning, by speaking, first, of the employment of purgatives: he is a bold purger, and, in the selection of his remedies, prefers the compound powder of jalap, or the neutral salts, to doses of calomel; he advocates their employment until natural stools are procured, and checks the disposition to constipation by smaller doses of the same medicine. With regard to blood-letting, our author leads us to believe that a prejudice against its general or vigorous employment exists, in this part of India, among the older practitioners; and he adds that, though he has frequently had occasion to blame himself for its omission, he never had reason to regret the employment of the lancet: he recommends it, therefore, to be practised in *every case*, unless some peculiar circumstance forbids it; at the same time, he inculcates a caution with respect to the quantity of blood to be taken away, which he in general thinks should be moderate, and rather calculated to prevent the general tendency to congestion, in the liver especially. Particular local pain in the head may also demand the abstraction of blood from the temples; and tenderness in the region of the liver may call for the application of blisters.

*Cold affusion* is the next remedy spoken of by our author, which is used much too sparingly in India; the reasons for which are certainly not very cogent, if they be no other than those our author has surmised. The rule for the employment of this powerful remedy is thus given:

"Whenever I have visited a patient in fever, and found him with a heat steadily and considerably above the natural standard, I have immediately had him brought into the hospital verandah, and had several pots of water (rendered colder by the addition of a handful of salt to each,) dashed over him; and, after being rubbed dry, he was returned to bed, when this practice was generally succeeded by profuse sweating. After this, the employment of a purgative has generally prevented the disease from gaining any permanent footing, and the patient has often, in the course of eight-and-forty hours, been returned to his duty. Whenever a return of the extreme heat of skin, and an exacerbation of the febrile symptoms, have rendered a repetition of the affusion necessary, the patient has submitted to it, not only without reluctance, but with the greatest cheerfulness, and most perfect reliance on its beneficial effects."

It is only, however, in the early stage of fever that this remedy is admissible, before any of the abdominal viscera have become decidedly the seat of disease.

We feel ourselves called upon in this place to give our feeble support to these remarks of Dr. Ballingall: we have certainly not seen the exact fever which he describes, but, in the common continued fever of the south of Europe, so frequent in the latter end of summer and the beginning of autumn, we have ourselves had occasion to practise the cold affusion very largely, and we can safely declare that, whilst the benefits derived from its employment have been most striking, we are not aware of any evil consequence that has ensued from its use. In many instances, affusion of two or three pailsful of cold water has been sufficient to cut off the fever altogether: in such cases it is followed by immediate and profound sleep, and the patient, before perhaps delirious, with a hot parched skin, intense thirst, and dry tongue, has awoke calm, perspiring, and without fever. We have not found pain in the head to forbid the use of this remedy; but local pain in any part of the body, giving cause to suspect visceral congestion, is a sufficient reason for withholding it. The heat of the skin should also be steadily above the natural temperament, *without any moisture;* and, although it is more efficacious in the first days of the disease, we have seen it most decidedly useful when employed as late as the seventh or eighth day, under the restrictions above mentioned.

To return to our author. *Mercury* is the next remedy which he notices, and he is inclined to think that a liberal use of purging and blood-letting will, very generally, render its exhibition unnecessary; though this is not the common opinion of the practitioner in India. It, however, becomes indispensable if, after an attack of fever, there should be reason to suspect derangement of the liver, often denoted by frequent relapses, and then assuming the intermittent form. There is, however, nothing peculiar in what our author advances on this subject, and we therefore may be allowed to pass it over in silence.

*Emetics* are generally condemned by Dr. B. in the fevers of India. Vomiting, he says, when once induced, is seldom inclined to stop where we wish it, and often forms one of the most perplexing and troublesome symptoms of the disease. (p. 42.) The only reason that induced him to prescribe an emetic was the supposition of the stomach being overloaded with bile, or with any indigestible substance recently swallowed.

Lastly, some remarks are made upon the exhibition of *bark*, and in which we, from our own practical knowledge, entirely concur. If the fever has been completely overcome, bark is unnecessary, for the strength will revive as surely without it as with it: if, on the contrary, the foundation of some visceral affection be laid, bark can only increase the evil: when the fever assumes the intermittent form, this is almost always the case, and the exhibition of bark, if it check the paroxysm for a time, ultimately fails in producing a cure, which can only be achieved by overcoming the visceral obstruction or derangement, which is the *cause* of the ague, and the latter will then cease without the aid of bark. It has been our lot to be placed in situations where intermittent fevers were perpetually occurring, and where, if the cure had depended upon bark, it would have been impossible to have met with an adequate supply: in no one instance did we fail to arrest the progress of the disease by those remedies (mercury especially,) which had for their object the removal of the evidently deranged state of the hepatic organs. Those who have practised extensively in the fever of Walcheren will, we are confident, admit the justice of these remarks.

The next division of Dr. Ballingall's work is devoted to the consideration of *dysentery*, and is, upon the whole, the most important subject contained in it. Our author begins thus:

"This disease, as it occurs in India, differs materially from the definition of our celebrated English nosologist. Instead of the '*pyrexia contagiosa*,' which that definition leads us to suspect, and which is the first characteristic mentioned by Dr. Cullen, the dysentery of India often makes considerable progress, and has very seriously, perhaps irreparably, injured the intestinal canal, before any urgent symptoms of pyrexia become either distressing to the patient or conspicuous to his medical attendant; and, with regard to its contagious nature, it may be sufficient to observe that, amongst some thousand cases of this disease which I have seen and treated, no one circumstance has occurred tending to excite even a suspicion of its being propagated by contagion. The appearance of scybalæ, another striking feature of the disease, as described by some writers in Europe, is certainly a rare occurrence in India; and there are various other circumstances tending to show that the flux prevalent amongst the troops in that country bears but a remote similarity to the dysentery of our European nosologists.

"There is another series of appearances which, in my opinion, point out two distinct varieties of dysentery as it occurs in India: the one an acute disease, confined chiefly to the large intestines, and which, by some of the Indian practitioners, has been not inaptly termed Colonitis, a term not necessarily implying the existence of a flux, but corresponding extremely well with the appearances on dissection; the other, a more chronic form of disease, and more extended in its site, has been, with some propriety, denominated the hepatic flux. I shall endeavour, throughout this paper, to keep these two varieties distinctly in view, convinced that this will be the means of leading to a more correct conception of the nature of Indian fluxes, and will, it is to be hoped, be the means of suggesting a rational and discriminating practice.

"On the first arrival of European troops in India, the colonitis, or inflammation of the large intestines, is the disease which principally proves destructive to them; and it is to this form chiefly I allude, in saying that the disease often makes considerable progress before any urgent febrile symptoms take place. This suggests the necessity of extreme caution in deciding on the cases of soldiers; and, whenever the smallest doubt arises about the correctness of a patient's statement, it will be prudent to order him immediately into hospital, and to have his evacuations examined, so as, on the one hand, to prevent ourselves being imposed on, and, on the other hand, to insure speedy and effectual assistance to every man really ill. There is, perhaps, no disease in which the necessity of an adherence to the established maxim '*obsta principiis*' is more conspicuous than in this, and perhaps none in which, after a certain stage, the efforts of medicine become more completely unavailing." (p. 45—47.)

Our author confesses himself at a loss to account for the frequent occurrence of this disease on the first arrival of a European regiment in India, and, leaving this speculation as unprofitable, he proceeds to describe the common mode in which this disease attacks the patient; the commencement being that of common diarrhœa, gripings in the bowels, frequent calls to stool, and strong inclination to strain. The evacuations are copious, fluid, and without any peculiar fœtor; they are sometimes streaked with blood. The pulse is seldom much altered; but the thirst is intense, the prostration of strength great, and the appetite gone. To these symptoms, pain in the hypogastrium, and in one or both iliac regions, succeed, with tension, fulness, and tenderness on pressure. The evacuations become more frequent, though less copious, consisting only of blood and mucus; suppression of urine, and tenesmus, now ensue, and the thirst is unconquerable; the tongue varies,—sometimes florid, smooth, and glossy, at others white and furred; the skin either very hot or in a state of extreme perspiration; the pulse often little affected, but, when it is, without any great increase of velocity, full and bounding, with a peculiar thrilling sensation under the finger, extreme danger is denoted. As the scene draws to a close, the stools become extremely fetid, mixed with shreds of membrane and purulent matter; protrusion of the gut frequently takes place; and death, preceded by delirium, hiccough, and cessation of pain,—in short, by the symptoms of gangrene,—relieves the sufferer from this loathsome state of existence. The disease completes its course sometimes within a week; at others, it lasts upwards of three weeks, but seldom longer.

Our author next proceeds to describe the more chronic form of disease, usually called the *hepatic flux*. This is more frequently found in men who have been resident some time in India, who are less prone to inflammatory affections, but more so to irregular or inordinate secretions of bile. In the first symptoms there is a close resemblance to the disease above described, but the stomach appears to suffer more: nausea, a disagreeable taste in the mouth, a loaded tongue, and a quickened pulse, are noted as generally present. After some days, the stools become of a whitish colour, frequently mixed with portions of half-digested aliment; loathing of food, hiccup, and bilious vomiting, now become highly distressing; the thirst becomes more and more urgent; emaciation rapidly proceeds; and these symptoms, modified by constitution and circumstances, often continue for weeks, or even months; and, when the patient dies, it is usually in consequence of the occurrence of an abscess in the liver, or of ulceration and mortification in the colon.

Then follows an account of the appearances on dissection in the cases of colonitis, and which, in a great proportion of cases, consist solely of inflammation of that part of the intestinal tube situated below the valve of the colon, without the slightest trace of disease in the liver. We shall not stop to enumerate those appearances, it is sufficient to say, that they are almost exclusively confined to the great intestines, (to the sigmoid flexure of the colon especially,) and that they exhibit every shade of inflammation, from slight redness to complete mortification, ulceration, and so great a thickening of the internal coat, as occasionally much to diminish the natural dimensions of the canal. Scybalæ are very seldom met with.

We now come to the means of cure.

"From the preceding detail of symptoms, and the appearances met with on dissection,—from the slightness of the constitutional symptoms, and the violence of the topical affection,—I have been led to consider the colonitis, or acute form of flux, as much more of a local disease, and of course more under the power of local remedies, than has generally been imagined; to look upon it as a disease confined almost exclusively to the large intestines: an inflammation, in short, of this part of the canal, tending rapidly to mortification, and having, in a great proportion of cases, little or no connexion with disease of the liver. In many of the most desperate cases of this disease, there has been so little reason to suspect an affection of this latter organ, from the existing symptoms during the life of the patient, and so trifling have been its diseased appearances on dissection, that I have been almost tempted to look upon them as the result of a secondary affection, communicated to the liver from its contiguity to the hepatic flexure and transverse arch of the colon,—to look upon them as an effect rather than a cause of the disease.

"But, even allowing that a diseased action of the liver, and a vitiated state of the biliary secretion, have always preceded the attack of colonitis, and have even been, in some measure, the cause of the latter affection, still, in the state we meet the disease, the effect appears greatly to have outrun the cause; they bear no adequate proportion to each other: and it is too much to expect that, by taking away the one, the other will cease. It would be of little consequence to exhibit remedies calculated to restore a healthy action to the liver, while the patient was dying of mortification of the colon." (p. 66—68.)

In order to combat these symptoms, our author lays particular stress upon topical bleeding, fomentations, blisters, and anodyne injections, which, instead of considering as mere auxiliaries, he is induced to regard as essential to the cure. Of general blood-letting, under particular circumstances, he has, however, a good opinion; but his experience on this point is not extensive, and his expressions somewhat vague. Purgatives he is unfriendly to: he advises their administration on the first admission of a patient, to ascertain whether the bowels are loaded with fæcal matter or not; but, viewing the disease in the light above mentioned, he of course considers their repetition unnecessary, if not hurtful; and the same objections apply to emetics. Occasional reasons may exist for their administration, which the practitioner can easily understand; but they are not useful as a mode of cure, in either form of dysentery. Of sudorifics, Dr. B. speaks with high approbation and confidence: the form he prefers is the union of opium and ipecacuanha, and which was employed first by Mr. ABERCROMBY, when surgeon to the 36th regiment. It was usual to give several grains of solid opium, (in what period of time is not specified,) and to follow its exhibition with two or more ounces of the infusion of ipecacuanha. Dover's powder, antimony, and opium, were likewise occasionally employed, the choice depending upon the greater or less degree of irritability of the stomach. It must be remarked, that smaller doses of these medicines are required in India than it is usual to prescribe in colder climates. Warm baths are found to be highly efficacious; but of mercury his opinion is unfavourable in this disease, and he is borne out by the result of his dissections, as well as by the result of his own practice in Prince of Wales's Island; and he adds—

"The dissection of every subject who died of dysentery in the regimental hospital at Penang, (with one solitary exception,) proved the disease to consist entirely in an inflammatory affection of the large intestines, without a trace of disease in the structure of the liver.

The consideration of this circumstance, and the reflection that mercury, when given for the cure of other complaints, is apt to produce some of the very symptoms which are here so distressing, soon induced us to be more circumspect in the use of this powerful remedy. Pains in the bowels, griping, and tenesmus, mucous and even bloody stools, are not very unfrequent occurrences from the extensive use of mercury; and this medicine is therefore, in my opinion, not very likely to be useful in removing them. I can readily conceive, and indeed know, that, in cases of a protracted disease, where the discharges from the intestines degenerate from pure blood and mucus, and become more of a diseased nature, that there is no remedy so much to be depended upon for the restoration of healthy secretions; but, in the pure inflammatory complaint I am now speaking of, mercury can seldom be useful. (76—77.)

We omit his remarks upon topical bleeding and blisters, because we have urged their employment strongly above, and proceed to notice our author's remarks upon injections, and which he is induced to consider as a very essential point in the cure,—alleviating the tenesmus, diminishing the frequent calls to stool, and lessening the profuse discharges of blood and mucus. In some instances, decoctions of bark, solutions of acetate of lead or sulphate of zinc, have been thrown up cold: in more moderate cases, the common anodyne injection, composed of rice, gruel, and tincture of opium, administered three or four times in the day.

In the chronic or hepatic form of dysentery, the constitutional origin of the disease is to be considered marking a derangement of the functions of the glandular viscera of the abdomen, as well as of the intestinal canal; and it is to this form of disease that the employment of mercury is warmly advocated by our author, and the form he recommends is the blue-pill; though he observes that every practitioner in India has a partiality for some particular preparation of this medicine, but, if irritability of the stomach or bowels exists, he of course prefers its external use; or calomel combined with opium may be given without increasing that effect. Whatever form be used, it is to be carried to the extent of producing ptyalism, which is to be kept up, without intermission, until natural secretions return, and the stools resume a healthy appearance. Dr. B. is not friendly, however, to profuse ptyalisms.

In this form of flux, purgatives are said to be useful in every stage of the complaint: in the choice of these remedies, we are to be guided, in some degree, by the feelings, or even prejudices, of the patients; and it may easily be conceived that particular symptoms may require various other remedies not mentioned by our author. Swathing the abdomen in flannel is a practice decidedly beneficial, and never to be omitted.

With regard to regimen, a few words only are said. In the acute form of the disease, the antiphlogistic plan, with abstinence from wine, must be strictly adhered to. In the chronic form, the choice of the patient, with regard to the kind and quality of the aliment, may be consulted. In conclusion, Dr. Ballingall observes, that though, under the general denomination of dysentery, we have two distinct forms of disease prevalent in India, yet they are frequently blended together, and met with in every possible variety of combination; in which case the practitioner must be guided, in his *methodus medendi*, by the particular circumstances of the case.

Of the two remaining divisions of this volume, it is not our intention to enter into an examination. The short section devoted to *liver complaints*, though containing good rules of practice, does not present to us any feature of remarkable interest or novelty; and the Essay on *Syphilis*, although deserving of commendation at the time it was written, has lost much of its value, in consequence of the subsequent discussions that have taken place on the subject. It is too short a paper to embrace one-tenth part of the question, but, as far as it goes, it is well calculated to restore confidence in the rational employment of mercury in that class of diseases; a confidence, which it never was the intention of those who instituted the non-mercurial treatment to overthrow, but merely to regulate,—to show that there are circumstances in which the wiser and more prudent course is to withhold that remedy for a time,—to teach us that there are certain constitutions in which it operates as a poison solely,—that all cases of syphilis need not be subjected to precisely the same treatment, in kind or in degree,—and, in doing this, it has performed a most essential service, and placed the practice in syphilis upon a scientific basis, instead of that fatal course, too nearly allied to empiricism, which devoted every ulcer on the genitals indiscriminately to a certain quantity of salivation, and a tedious confinement.

There are five Appendices to the volume: the first contains a register of deaths of the second battalion of the 1st Foot, from 1808 to 1813; the second contains cases of fever; the third, select cases of dysentery; the fourth, of liver complaints; and the fifth is a general statement of the deaths of the European troops, at the principal stations of the Madras establishment, from January, 1807, to October 31, 1808.

In analysing Dr. Ballingall's volume, if we have indulged in but few remarks of our own, we have been induced to do so principally because (although we have, unfortunately, had ample experience, upon a large scale, in the diseases of which he treats,) we do not profess to know any thing of the climate in which he practised. His remarks bear the stamp of good sense and sound doctrine, and we recommend them to the perusal of those, especially, whose destinies call them to follow the medical profession in the Eastern hemisphere. There is, it must be confessed, notwithstanding the alterations our author has made in this edition of his work, a looseness of expression occasionally observable; but candour and sincerity are obvious throughout, and much must be conceded to the author, who, in despite of the fatigues of service, the accumulation of sick, and the perpetual calls upon his time and attention, presents us with so valuable a collection of facts and observations.

---

### DIVISION II.

### FOREIGN.

ART. III.—*De Nervi Sympathetici Humani, fabrica, usu, et morbis; Commentatio Anatomico-Physiologico-Pathologica, &c. &c.* Auctore J. F. LOBSTEIN, &c.—Parisiis, 1823.

THIS work, which is executed in a manner highly creditable to the distinguished author, is devoted to the consideration of the sympathetic nerve, comprising its anatomy, physiology, and pathology; the dissertation being divided into three corresponding sections.

It is almost unnecessary to remark, in these times, that those anatomists were much deceived who attributed ganglia to the sympathetic alone, denying them to other nerves: it is now sufficiently established, that the nerves of the spine, as well as those of the head, possess ganglia, although they are wholly unconnected with the functions of digestion, nor belonging in any way to the system of BICHAT, in which the ganglionic nerves were separated from those of the brain and spinal marrow.

In the first section, or division, are considered the seat, course, branches, and anastomoses; and next the disposition of the plexuses, and the structure of the ganglia; having premised some observations on the development of this system in the fœtus. The great sympathetic is considered by LOBSTEIN as one continuous nervous cord, running from the head to the pelvis, swelling into many ganglia, but never, or at least very rarely, distinctly interrupted. It is said to have two extremities, one in the head and the other in the pelvis; by which general terms the questions of its origin and termination are altogether avoided. Another question, which appears to us equally idle, is likewise judiciously passed by: it relates to the branches which are *received*, and those which are *sent off:* the only division adopted by our author is the practical one of external and internal branches; the former being those which are more remote from the axis of the body, and connect the sympathetic with the spinal nerves; the latter those branches which are distributed to different organs, and always accompany the vessels. Before commencing his description, the author informs us that he has traced the sympathetic in many bodies, and has always found some variety with respect to the origin and course of the branches; the description given in the work before us being taken from a young man, aged twenty-four years, who had been imbecile from his birth, but who had the nerve in question particularly well developed.

It would be alike tedious and unprofitable to our readers were we to follow this intelligent anatomist through his elaborate and minute description of the sympathetic nerve, in all its numerous ganglia and ramifications, which are severally and individually discussed; we shall therefore pass on to the second part of his discourse, which relates to the history of this nerve, as given by others: and first with regard to its *cephalic* extremity. Bock, in a splendid letter written in German,* describes the sympathetic as ascending into the carotid canal from the cervical ganglion; it is then divided, according to the author, into two branches, which, through the medium of the plexiform ganglion, anastomose with the Vidian nerve, with the abductor, and the motor oculi; some minute twigs are added, which enter the coats of the carotid artery. The same is asserted by Ribes,† who further states that, after washing the brain and nerves, he detected a fassiculus given off by the nervous investment of the carotid artery, accompanying the ophthalmic artery in all its branches, and penetrating the internal eye with the central artery of the retina. He likewise adds, that the ophthalmic ganglion receives a branch from the sympathetic, from which follows the sympathy between the retina and the nerves of the iris. Cloquet, apparently guided by these remarks, places the origin of the sympathetic in the orbit and interior of the eye; admitting an anastomosis between the superior cervical ganglion and the ophthalmic nerve.‡ It is to be observed, that the ramuli of the sympathetic nerve in the canal of the carotid are not of recent discovery, being explicitly described by Winslow, as accompanying that vessel into the head. The most minute detail, however, seems to be that of Fontana,§ who admits twigs of three different orders as situated on the carotid canal, and belonging to the sixth pair of cerebral nerves: the first encircling this nerve in the form of a soft membrane; the second separated from, but placed near it, unite with the carotid artery;

* *Beschreibung des fünften Nervenpaars und seiner Verbindungen mit andern Nerven, &c.* 1817.

† *Memoires de la Societe Medicale d'Emulation*, tom. vii. et viii.

‡ *Traité d'Anatomie descriptive*, tom. ii. p. 689-90.

§ *Precis d'une Dissertation de* M. Girardi *et des Recherches de* M. Fontana, *sur l'Origine du Nerf Intercostal.—(Journal de Medecine Chir. et Pharm.* par Bacher. Tom. xciii. p. 53—9.)

the third adhere to the above-mentioned nerve. He saw branches going to the ophthalmic of WILLIS, to the superior maxillary, to the coats of the carotid, to the pituitary gland, and to the orbit; and, lastly, five twigs communicating with the vidian nerve.

We have dwelt upon this point the rather, as we are inclined to suspect that anatomists sometimes favour us with a description of appearances which have no existence in nature, and are, in fact, the product of the steeping, washing, and other operations, employed with a view of rendering the parts more distinct: this they certainly do, but is it not sometimes at the expence of truth? Hear Lobstein. "My dissections, which were begun long ago, and have been frequently repeated, do not confirm the abundance *(copiam)* of branches described by these various writers. It is true I have frequently detected pellucid filaments of a gelatinous nature, joining surculi of the sympathetic with the motor oculi and other nerves; but, viewed through a microscope, they could not be regarded as true stamina of nerves. The method of those who endeavour to evolve and render nervous branches distinct by washing, is therefore to be distrusted; for, by this means, the common cellular texture is reduced into white flocculi, which are easily mistaken for nervous twigs."

Another interesting question relates to the anastomoses described by Professor JACOBSON,* as existing between the fifth pair, the glosso-pharyngeus, and sympathetic. According to this anatomist, the superficial vidian nerve received into the fallopian fissure is composed of three ramuli, whereof the first joins the fascial, and the two others, included in their proper canals, penetrate the tympanum, in order to effect the anastomosis above mentioned. It is added, that this anastomosis is never wanting, affords no anomalies, and is present in all animals. Ribes, however, calls in question this account;† while Cloquet admits the nerves described by Jacobson, without alluding to their discoverer. Either such nerves do exist, or they do not. We cannot but feel mortified with the contradictory testimony, given by authors of equal authority, with regard to what ought to be matter of fact, not opinion.

It is from the consideration of these circumstances, and from having been frequently struck with similar contradictions, that we are led to view with distrust anatomical descriptions so minute—that not one in ten can ever find the parts. Jacobson states, that the superficial vidian nerve is composed of three ramuli, which enter the bone in separate and proper canals;

---

* *Acta Regiæ Societatis Hafniensis Medicæ.* Hafniæ, 1818.

† *Dictionnaire des Sciences Medicales,* tom. lvi. p. 151.

while it is added, that this distribution affords no anomaly, (*" nullam anomaliam suppeditare."*) KILIAN, on the other hand, asserts* that a nervous twig from the glosso-pharyngeus sometimes arrives at the tympanum, but more frequently terminates in the substance of the pars petrosa, *never* being associated with the superficial vidian, and *never* entering the bone by three distinct canals. " Nunquam vero nervo Vidiano superficiali associari; hiatum fallopii semper esse simplicem, et nunquam in tres canaliculos pro recipiendis tribus filamentis nervosis esse divisum!" It is further added, that no branches from the sympathetic enter the tympanum.

We now come to the description given by the learned author before us:

" A twig ascending from the first cervical ganglion, or arising from the trunk of the sympathetic before the formation of the ganglion, enters its proper foramen, situated at the first flexure of the carotid canal. This (foramen) leads to a canal which is cut out in the bone, receiving the twig above mentioned. This (twig), creeping through the substance of the bone, divides into two branches, which mutually diverge from each other; of which, the former, being finer and anterior, penetrates into the cavity of the tympanum, runs downwards upon the promontory, and joins with a branch from the petrous nerve. The other, a little thicker, is directed towards the posterior part of the tympanum, in a bony canal, and is united with another twig from the nerve above mentioned. From this union arises a branch which goes out through the foramen, which is near the receptacle of the glosso-pharyngeus, and inosculates with this nerve."

The irregularity of the sympathetic with regard to its course and distribution, are too well known to require much notice here. That it is totally interrupted, as asserted by HALLER, and more particularly by BICHAT, is not so unequivocally shown, and indeed is denied by our author; in which opinion he is supported by WRISBERG,† WEBER,‡ and other distinguished anatomists.

The next interesting part of the discussion before us relates to the connexion between the nerves and vessels. The lymphatics and veins are generally stated to receive no nerves, if we except the branches of the vena portæ, the pulmonary, and the jugular; while the arteries have an obvious association with the nerves. The first connexion consists in the larger arterial trunks being surrounded by nervous twigs, without any coherence: thus the vertebral artery is embraced at its origin by branches from the cardiac plexus, so numerous as to resemble an investing membrane. A very different relation, however,

* *Anatomische Untersuchung über des neunte Hirnnervenpaar.*—Pesth, 1822, p. 71.

† *Observ. Anat. de Ganglio plexuque semilunari, &c. Comment.*—Gotting. vol. ii.

‡ *Anat. Compar. Nervi Sympathetici.*—Lips. 1817.

obtains in almost all those branches of arteries which are accompanied by ganglionic nerves. In these cases, numerous plexuses follow the vessels, closely united to their tunics, and penetrating the various organs along with them, become soft, and finally disappear from the eye. Examined with the assistance of a microscope, they are described by Lobstein as follows:—The ultimate filaments of the nerves accompanying arteries divide into numerous little fibres, which, closely applied to each other, form almost the whole of the cellular mesh *(rete cellulosum,)* which surrounds these blood-vessels; and in which are seen white streaks. The author states, that he has frequently confirmed this observation, by following the nervous filaments which accompany the branches of the external carotid. The cellular investment of the lingual and other arteries, when subjected to a microscope magnifying sixty times, appeared to be nothing but the mesh above mentioned, in which streaks of a white colour run in the manner described, and being entirely distinct from common cellular texture; for example, from that investing the sterno-hyoideus muscle. On this point our author is explicit in his opinion, that a nervous cellular tissue is given to the arteries and branches of nerves, totally distinct and distinguishable from ordinary cellular texture.

The question next considered is, whether all the nerves of the arteries vanish in this cellular web, or, some perforating the coats of the vessels, are distributed on their substance? In answer to this, he informs us that he has seen two small nerves, arising from the superficial cardiac of the right side, perforate the arch of the aorta, before the origin of the arteria innominata, and lose themselves in its middle tunic; and that he has been able to demonstrate, in the fœtus, a branch perforating the cellular membrane of the aorta, behind the origin of the left subclavian, and disappearing in its middle coat. Walter and Lucoe have described similar appearances.

In considering the distribution of the sympathetic, as described by Lobstein, an important difference is to be remarked between the exterior, or communicating branches, and the interior, or those which go to the various organs. The former never surround arteries, although they sometimes accompany them, and many are found without being associated with these vessels. He has never found arterial branches going along with anastomosing twigs of the cervical or dorsal nerves, (this remark, however, does not apply to the nutrient vessels of the nerves;) nor are there anastomosing nerves ever subdivided into branches, before they have inosculated with the intercostal nerve. The latter, on the other hand, give off branches which hasten to the organs, and always accompany arteries. The former have invariably the same colour, density, and general structure, whether they be examined in the neck, the back, loins, or sacral region; the latter vary in their nature and appearance. Thus, in the carotid canal, we find twigs which are red, fine, and flat rather than cylindrical; in the neck we find branches which are thick, soft, red, and in some measure pellucid; and, again, filaments which are fine and white. The differences in the abdomen are still more remarkable, and, indeed, too numerous to be detailed.

The ganglia likewise differ essentially from each other. Take three of the most important,—viz. the ganglion cervicale supremum, the ganglion thoracicum primum, and the ganglion semilunare: the first of these is longer, softer, and redder, than the others; the second is harder, and of a less deep-red colour; whilst the third is the hardest of all, and varies so much in figure as scarcely to be alike in two different individuals, resembling internally the human cutis, or salivary glands, when in a state of health.

[To be continued.]

